# Mathematical modeling of population structure in bioreactors seeded with light-controllable microbial stem cells

**DOI:** 10.3934/mbe.2020415

**Published:** 2020-11-13

**Authors:** Dane Patey, Nikolai V. Mushnikov, Grant R. Bowman, Rongsong Liu

**Affiliations:** 1 Department of Mathematics and Statisitics, University of Wyoming, 1000 E. University, Laramie, WY 82071, USA; 2 Department of Molecular Biology, University of Wyoming, 1000 E. University, Laramie, WY 82071, USA

**Keywords:** mathematical models, population dynamics, cell division, mutant

## Abstract

Industrial bioreactors use microbial organisms as living factories to produce a wide range of commercial products. For most applications, yields eventually become limited by the proliferation of “escape mutants” that acquire a growth advantage by losing the ability to make product. The goal of this work is to use mathematical models to determine whether this problem could be addressed in continuous flow bioreactors that include a “stem cell” population that multiplies rapidly and could be used to compete against the emergence of cheater mutants. In this system, external stimuli can be used to induce stem cell multiplication through symmetric cell division, or to limit stem cell multiplication and induce higher production through an asymmetric cell division that produces one stem cell and one new product-producing “factory cell”. Our results show product yields from bioreactors with microbial stem cells can be increased by 18% to 127% over conventional methods, and sensitivity analysis shows that yields could be improved over a broad range of parameter space.

## 1. Introduction

Many industries use microbial cells as factories for making chemical products. Pharmaceutical companies use genetically modified bacterial strains to produce a range of biological therapeutics, including several “blockbuster” drugs that exceed one billion dollars in annual sales [1], and similar biomanufacturing approaches are used to produce health supplements and other bioactive molecules [2]. Microbes are also used for making digestive enzymes as additives to commercial detergents [3, 4] and producing cosmetic ingredients [5], and there is strong interest in using microbes to produce biofuels and other chemical products at industrial scales [6, 7]. In most cases, microbial strains must be genetically engineered in order to create large amounts of product. Unfortunately, the strategy of changing the organism to increase production is inherently associated with forces that work against that goal [8, 9]. This is because efficient product synthesis almost always comes with a trade-off in cell growth, as the energy required for synthesizing the desired product is drawn away from biological pathways that would otherwise be used for cell growth [10, 11]. In some cases, the desired product is toxic to the cells that are forced to produce it, and this further reduces the factory cells’ growth rate [12]. A highly significant down-side to slower cell growth is that these conditions favor the emergence of “escape mutant” cells that lose the ability to produce product due to random mutation [13, 14]. Because the escape mutants divide faster than factory cells, they will ultimately dominate the cell population and spoil production.

Lokta-Voterra competition models [15–17] have been widely used to model the competition between two species. In cellular level, mathematical models have also been used to describe the mutations, onset, progression and immune competition among cancel cells [18–20]. The goal of this study is to use mathematical modeling to determine whether production could be increased or prolonged in a continuous flow bioreactor by including a specialized cell type that is hereafter referred to as a “stem cell”. Without stem cells in the tank, because the growth rate of mutant cells are much faster than the factory cells, depending on the growth rates of these two types of cells, mutation rates, and other factors, the mutant cells will dominate and factory cells will die out sooner or later. Stem cells are biologically differentiated cells that do not create product, and on this basis they are distinguished from actively producing factory cells. Stem cells do not experience the metabolic burden associated with product synthesis, and therefore divide at the same rate as escape mutants that arise spontaneously in the population. Importantly, the population distribution of the system can be controlled with an external switch that imparts different modes of cell division. Under one mode, stem cells divide asymmetrically into one new factory cell and one regenerative stem cell. Under a second mode, stem cell division creates two stem cells, thereby increasing the relative population of this cell type. A question is whether the system can be controlled in a manner that supports a large population of factory cells and also maintains a rapidly dividing population of stem cells that effectively compete against the expansion of the escape mutant population.

**Figure 1.**
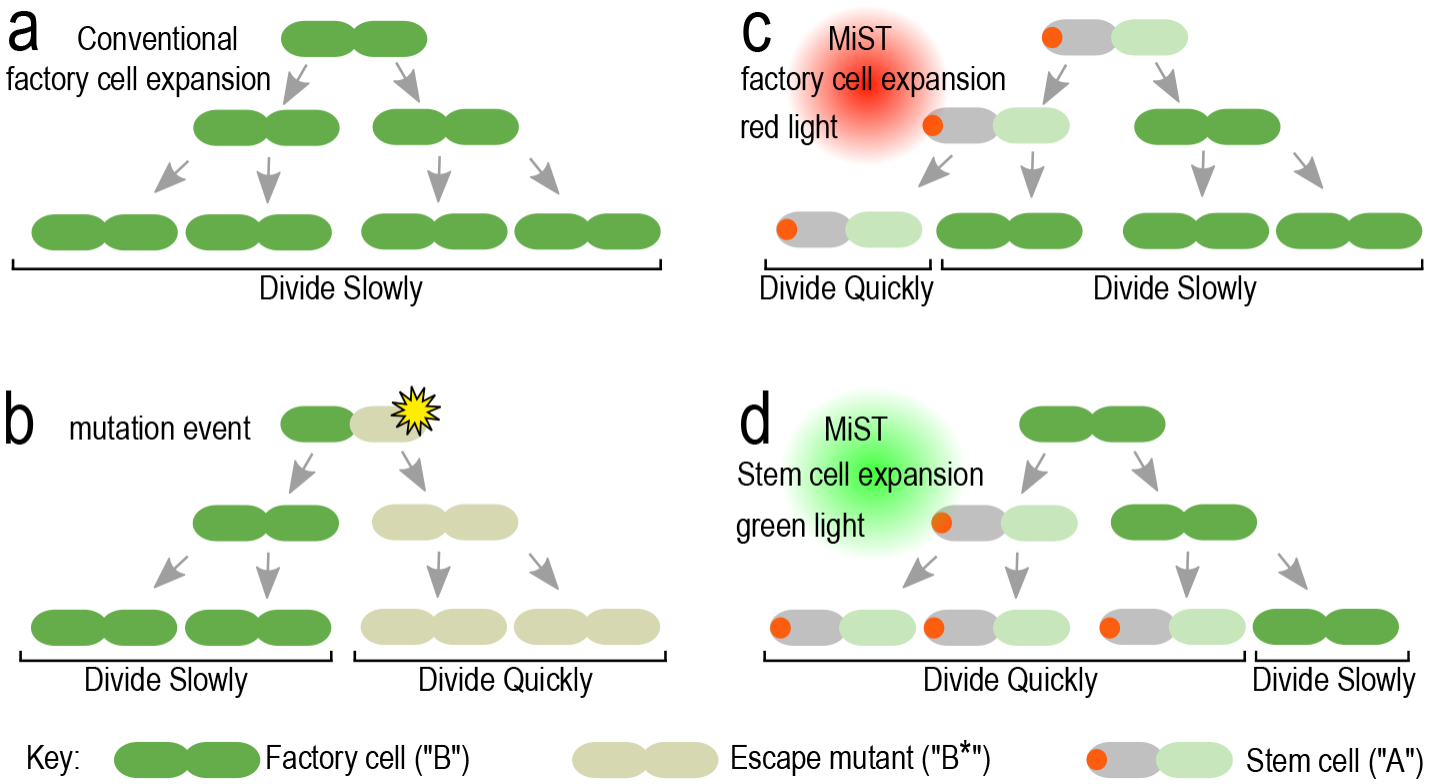
Patterns of cell division and cell fate in conventional and MiST cultures. (a) In conventional E. coli cultures, cell division is essentially symmetrical, resulting in daughter cells with equivalent behavior. Due to biosynthetic burden, engineered factory cells grow slowly as they generate product. (b) Spontaneous mutations can inhibit product synthesis, relieving the biosynthetic burden. These “escape mutants” divide relatively rapidly and overpopulate the culture. (c) MiST culture under red light conditions. Stem cells divide asymmetrically, producing one stem cell and another that differentiates into a factory cell. Factory cells divide symmetrically. Compared to factory cells, stem cells divide relatively quickly because they do not experience biosynthetic burden. (d) MiST culture under green light conditions. Stem cells divide symmetrically into two stem cells. Factory cells divide asymmetrically, producing one stem cell and another that retains factory cell identity.

**Figure 2.**
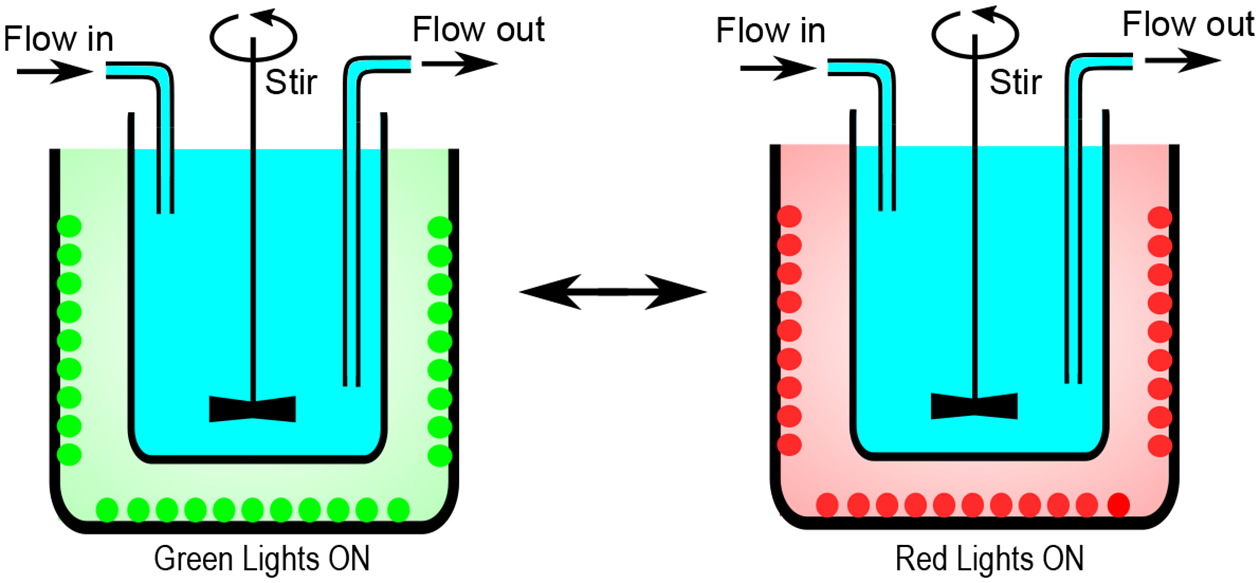
Conceptual diagram of a light-controlled continuous flow bioreactor for controlling MiST cultures. Cells in the culture vessel (shown here with transparent walls) can be exposed to red or green colored light.

The models presented in this work are intended to serve as a predictive tool for the population dynamics of microbial stem cells, factory cells, and mutant cells in an industrial bioreactor. The molecular and genetic mechanisms underlying the “Microbial Stem cell Technology” (MiST) upon which the models are based have been described [21]. Briefly, the system works by controlling the expression of a synthetic protein (YmP), which self-assembles into a single, asymmetrically localized geometric cue that is positioned at one end of a rod-shaped cell (called a cell pole). When a cell with a polar YmP cluster divides, one daughter cell inherits the pole-localized YmP cue while its sibling does not. Cells that inherit YmP are programmed to maintain stem cell fate whereas those that lack YmP differentiate into factory cells. Our models simulate the technique of using red or green light as stimuli for regulating the expression of YmP as a method for controlling stem cell or factory cell fate [21]. For the purpose of simplifying terminology in our mathematical expressions, we refer to stem cells as “*A* cells” and factory cells and “*B* cells”. Cells that have acquired mutations that block product production are denoted with astersisks, as in A* or B*. Figure 1 shows an illustration of four different cell division processes using conventional division, mutations, MiST red light, and MiST green light scenarios. Figure 2 describes the conceptual bioreactor switching between the two types of light cycles.

We will begin by describing the mathematical models for standard cell division with mutation in the section 2.1. Then we will describe the mathematical models for the red light, green light, green light star scenarios which include asymmetric cell division along side cell mutation, red light and green light as well as red light and green light star scenarios in the section 2.2. After presenting all the models used, in the section 3, we demonstrate their validity in real world applications using numerical simulations and sensitivity analysis. We present the summarization and discussion in the section 4.

## 2. Model

The most dramatic difference between factory cells and mutant cells is the doubling time of mutant cells is much shorter than that of factory cells. We assume the vessel is completely homogeneous so the distribution of each cell population in the vessel is uniform. The vessel’s carrying capacity for cells, denoted *K*, is the same as the total volume of the vessel.

### 2.1. A mathematical model for standard cell division

**Figure 3.**
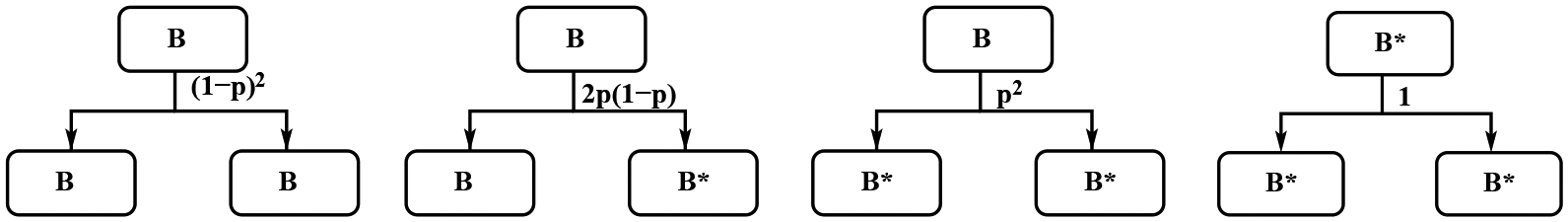
The dynamics of factory *B* cells and mutant B* cells in the standard cell division.

Let *B*(*t*) and B*(*t*) are the number of factory cells and escape mutant cells at time *t* in the vessel. Each factory cell will generate two cells through division, and we can name them A and B, respectively. During the division process, the generation of factory cells and generation of mutual cells are independent. The parameter *p* represents the possibility that one next generation cell is a mutant cell, noticing that *p* is a very small number, in the order of 10^−6^. Then the two next generation cells of a factory cell have scenarios, 1. both A and B are still factory cells with possibility (1 − *p*)^2^; 2. A is a mutant and B is a factory with probability *p* * (1 − *p*); and A is a factory cell and B is a mutant with possibility (1 − *p*) * *p*. Therefore the probability that one and only one of these two daughter cells is mutant is 2*p*(1 − *p*); 3. both are mutant cells with possibility *p*^2^, see Figure 3. If we add these three probability together, the sum is one. The mutant factory cells only produce mutant daughter cells, see Figure 3. The rate of changes for these cells can be described by the following system

(2.1) $$\{\begin{array}{l} \frac{dB(t)}{dt}=r_{b}B(t)C(t)-mB(t)\\ \frac{dB^{*}(t)}{dt}=r_{b}^{*}B^{*}(t)C(t)+c_{b}B(t)C(t)-mB^{*}(t) \end{array}$$

with initial value *B*(0) = *B*_0_, $B^{*}(0)=B_{0}^{*}$ and $C(t)=\left(1-\frac{B(t)+B^{*}(t)}{K}\right)$. Where *r*_*b*_ and $r_{b}^{*}$ are the intrinsic growth rates of factory cells and mutant factory cells, respectively, *K* is the carrying capacity for all the cells in the vessel, and *c*_*b*_ is the intrinsic growth rate of mutant factory cells due to the mutation of factory cells. Denote the doubling times for factory cells and mutant factory cells are *τ*_*b*_ and $\tau_{b}^{*}$. The parameter *m* is the continuous rate of the fluid out of the vessel, which is given units in terms of the volume of the vessel, *K*, per doubling time of mutant factory cells, $\tau_{b}^{*}$. Here, we assume that the factory cells and mutant cells have the same ability in term of competition.

We are going to derive the intrinsic growth rates. Assuming the cell populations are far less than the carrying capacity and there is no fluid out of vessel, in this case, the *C*(*t*) terms in the model (2.1) can be ignored. We have

(2.2) $$\{\begin{array}{l} \frac{dB(t)}{dt}=r_{b}B(t)\\ \frac{dB^{*}(t)}{dt}=r_{b}^{*}B^{*}(t)+c_{b}B(t). \end{array}$$

First, let us assume that at the beginning of the vessel, there are only factory cells and there is no mutant factory cell at all. Which gives *B*(0) = *B*_0_ and B*(0) = 0. Solving the first equation of (2.2), we can gain that

$$B(t)=B_{0}e^{r_{b}t}.$$

At its doubling time *τ*_*b*_, if there are initially *B*_0_ factory cells, there are (2(1 − *p*)^2^ + 2*p*(1 − *p*))*B*_0_ = 2(1 − *p*)*B*_0_ factory cells. Plugging *t* = *τ*_*b*_ into the above equation, we have

$$B_{0}e^{r_{b}\tau_{b}}=2(1-p)B_{0}.$$

Then, we can have that the intrinsic growth rate of factory cells with mutation is

(2.3) $$r_{b}=\frac{\text{ln}(2(1-p))}{\tau_{b}}.$$

Solving the second equation of (2.2), we have

(2.4) $$B^{*}(t)=c_{b}B_{0}\frac{e^{r_{b}^{*}t}-e^{r_{b}t}}{r_{b}^{*}-r_{b}}.$$

If B*(0) = 0, at *t* = *τ*_*b*_, the number mutant factory cells is B*(*τ*_*b*_) = 2*pB*_0_, see Figure 3. Plugging *t* = *τ*_*b*_ into Eq (2.4), we can get

(2.5) $$c_{b}=\frac{2p\left(r_{b}^{*}-r_{b}\right)}{e^{r_{b}^{*}\tau_{b}}-e^{r_{b}\tau_{b}}}.$$

Assuming initially in the vessel, there are only mutant factory cells and there is no factory cells, which are *B*(0) = 0 and $B^{*}(0)=B_{0}^{*}$. Similarly, we have the intrinsic growth rate of mutant cells directly from mutant cells is

(2.6) $$r_{b}^{*}=\frac{\text{ln }2}{\tau_{b}^{*}}.$$

The model (2.1) is a slightly varied Lokta-Voterra competition model [16]. As long as $r_{b}^{*}>r_{b}$, which is the reality in the experiments that the growth rate of mutant cells is always greater than that of the factory cells, and *r*_*b*_ > *m*, which guarantees that the factory cells can build up in the vessel, the mutant cells are always the stronger species and will be dominant and factory cells will die out at the steady state. In the following, we want to study the impact of introducing stem cells on the dynamics of the factory and mutant cells.

### 2.2 Mathematical models for the MiST cell division

#### 2.2.1. Red light scenario

**Figure 4.**
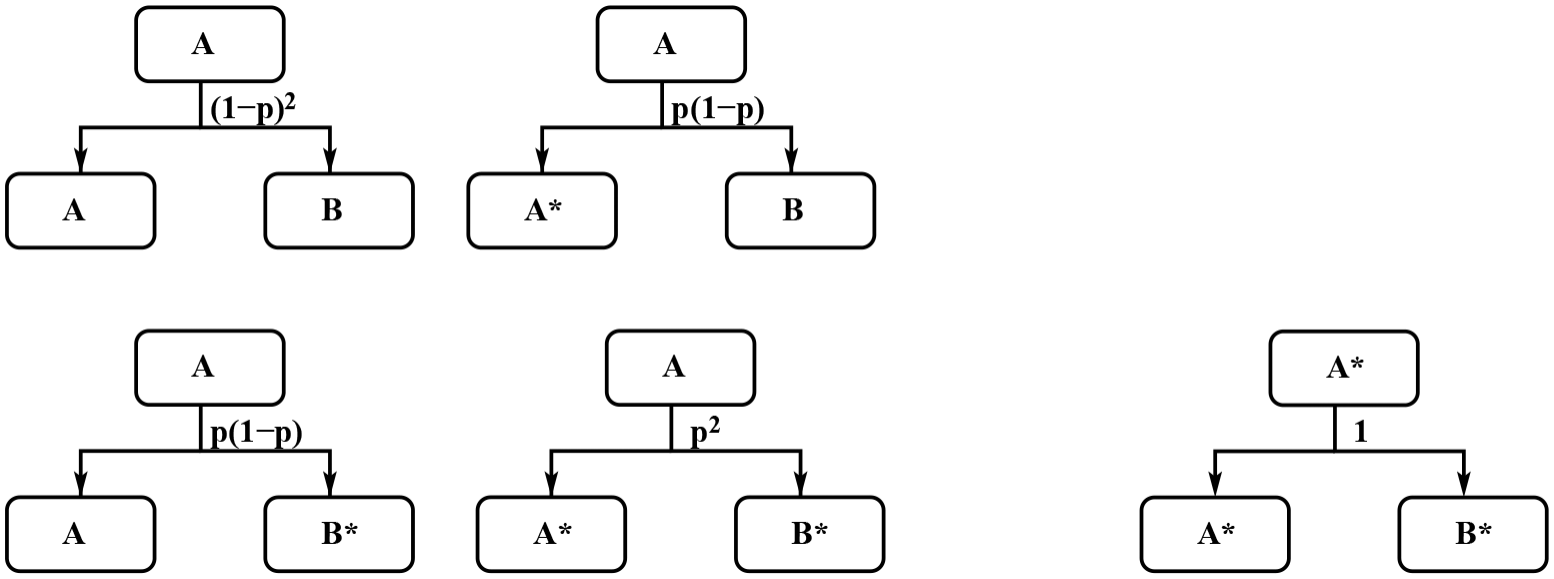
The dynamics of Stem *A* cells and stem mutant A* cells in the MiST cell division with red light scenario.

For the red light scenario case, in the vessel, there are factory cells and stem cells. The factory cells follow the same dynamics as the basic model, see Figure 3. We use the same notation *p* for the possibility that one of the daughter cells is mutant. Similarly, one stem cell will produce one stem cell and one factory cell with probability (1 − *p*)^2^, one mutant stem cell and one factory cell with probability *p*(1 − *p*), one stem cell and one mutant factory cell with probability *p*(1 − *p*), and one mutant stem cell and one mutant factory cell with probability *p*^2^. One mutant stem cell only produce one mutant stem cell and one mutant factory cell. The stem cells and mutant stem cells have the same doubling time as the mutant factory cells. The dynamics of stem cells and mutant stem cells in the red light case can be seen in Figure 4.

Let *A*_*r*_(*t*) and $A_{r}^{*}(t)$ represent the numbers of stem cells and mutant stem cells at time t, and *B*_*r*_(*t*) and $B_{r}^{*}(t)$ represent the numbers of factory cells and mutant factory cells at time t. The model in the red light case can be written as

(2.7) $$\{\begin{array}{l} \frac{dB_{r}(t)}{dt}=\left(r_{b}B_{r}(t)+c_{ab}A_{r}(t)\right)C_{r}(t)-mB_{r}(t)\\ \frac{dB_{r}^{*}(t)}{dt}=\left(r_{b}^{*}B_{r}^{*}(t)+c_{b}B_{r}(t)+c_{ab}^{*}A_{r}(t)+c_{ab}^{**}A_{r}^{*}(t)\right)C_{r}(t)-mB_{r}^{*}(t)\\ \frac{dA_{r}(t)}{dt}=r_{a}A_{r}(t)C_{r}(t)-mA_{r}(t)\\ \frac{dA_{r}^{*}(t)}{dt}=r_{a}^{*}A_{r}(t)C(t)-mA_{r}^{*}(t) \end{array}$$

with $C_{r}(t)=1-\left(B_{r}(t)+B_{r}^{*}(t)+A_{r}(t)+A_{r}^{*}(t)\right)/K$. The parameters *r*_*b*_, $r_{b}^{*}$, and *c*_*b*_ are defined in (2.3), (2.6), and (2.5), respectively. *c*_*ab*_, $c_{ab}^{*}$, and $r_{a}^{*}$ are the intrinsic growth of factory cells, mutant factory cells, and mutant stem cells due to the division of stem cells. *r*_*a*_ is the intrinsic growth rate of stem cells, and $c_{ab}^{**}$ is the intrinsic growth rate of mutant factory cells due to the division of mutant stem cells.

When there are enough resources for cells growth and there is no flow in and out from the vessel, the dynamics of the cells can be written as

(2.8) $$\{\begin{array}{c} \frac{dB_{r}(t)}{dt}=r_{b}B_{r}(t)+c_{ab}A_{r}(t)\\ \frac{dB_{r}^{*}(t)}{dt}=r_{b}^{*}B_{r}^{*}(t)+c_{b}B_{r}(t)+c_{ab}^{*}A_{r}(t)+c_{ab}^{**}A_{r}^{*}(t)\\ \frac{dA_{r}(t)}{dt}=r_{a}A_{r}(t)\\ \frac{dA_{r}^{*}(t)}{dt}=r_{a}^{*}A_{r}(t) \end{array}$$

with initial value *B*_*r*_(0) = *B*_*r*0_, $B_{r}^{*}(0)=0$, *A*_*r*_(0) = *A*_*r*0_, $A_{r}^{*}(0)=0$.

From Figure 4, we can see the number of stem cells is decreasing. At its doubling time $\tau_{b}^{*}$, the original one stem cell becomes (1 − *p*) stem cell. Which gives

(2.9) $$r_{a}=\frac{\text{ln}(1-p)}{\tau_{b}^{*}}<0.$$

Now, we need to derive the parameters *c*_*ab*_, $C_{ab}^{*}$, $C_{ab}^{**}$, $r_{a}^{*}$. From the third equation of (2.8), we have

(2.10) $$A_{r}(t)=A_{r0}e^{r_{a}t}.$$

Substitute *A*(*t*) into the fourth equation of (2.8), we get

$$A_{r}^{*}(t)=A_{r0}r_{a}^{*}\frac{e^{r_{a}t}-1}{r_{a}}.$$

At $t=\tau_{b}^{*}$, the number of $A_{r}^{*}\left(\tau_{b}^{*}\right)=pA_{r0}$, see Figure 4. Set $t=\tau_{b}^{*}$, from the above equation, we can solve

(2.11) $$r_{a}^{*}=\frac{pr_{a}}{e^{r_{a}\tau_{b}^{*}}-1}=-r_{a}>0.$$

From the first equation of (2.8), we have

$$B_{r}(t)=A_{r0}c_{ab}\frac{e^{r_{b}t}-e^{r_{a}t}}{r_{b}-r_{a}},$$

with the assumption *B*_*r*0_ = 0. When $t=\tau_{b}^{*}$, $B_{r}\left(\tau_{b}^{*}\right)=(1-p)A_{0}$. Set $t=\tau_{b}^{*}$ in the above equation, we get

(2.12) $$c_{ab}=(1-p)\frac{r_{b}-r_{a}}{e^{r_{b}\tau_{b}^{*}}-e^{r_{a}\tau_{b}^{*}}}.$$

From Figure 4, we can observe that the increasing rates of mutant stem cells and mutant factory cells due to the stem cells are the same. So we have

$$c_{ab}^{*}=-r_{a}.$$

Assuming *B*_*r*_(0) = 0, *A*_*r*_(0) = 0, we aim to find the value of parameter $c_{ab}^{**}$, the increasing rate of mutant factory cells only due to mutant stem cells. Solving the second equation of (2.8) with the assumption that *B*_*r*_(0) = 0, *A*_*r*_(*t*) = 0, $B_{r}^{*}(0)=0$, $A_{r}^{*}(0)=A_{r0}^{*}$, it gives

$$B_{r}^{*}(t)=c_{ab}^{**}\frac{e_{b}^{r_{b}^{*}t-1}}{r_{b}^{*}}A_{r0}^{*}$$

When $t=\tau_{b}^{*}$, $B^{*}\left(\tau_{b}^{*}\right)=A_{0}^{*}$. Setting $t=\tau_{b}^{*}$ in the above equation, we have

(2.13) $$c_{ab}^{**}=\frac{r_{b}^{*}}{e^{r_{b}^{*}\tau_{b}^{*}-1}}=r_{b}^{*}.$$

#### 2.2.2. Green light scenario

**Figure 5.**
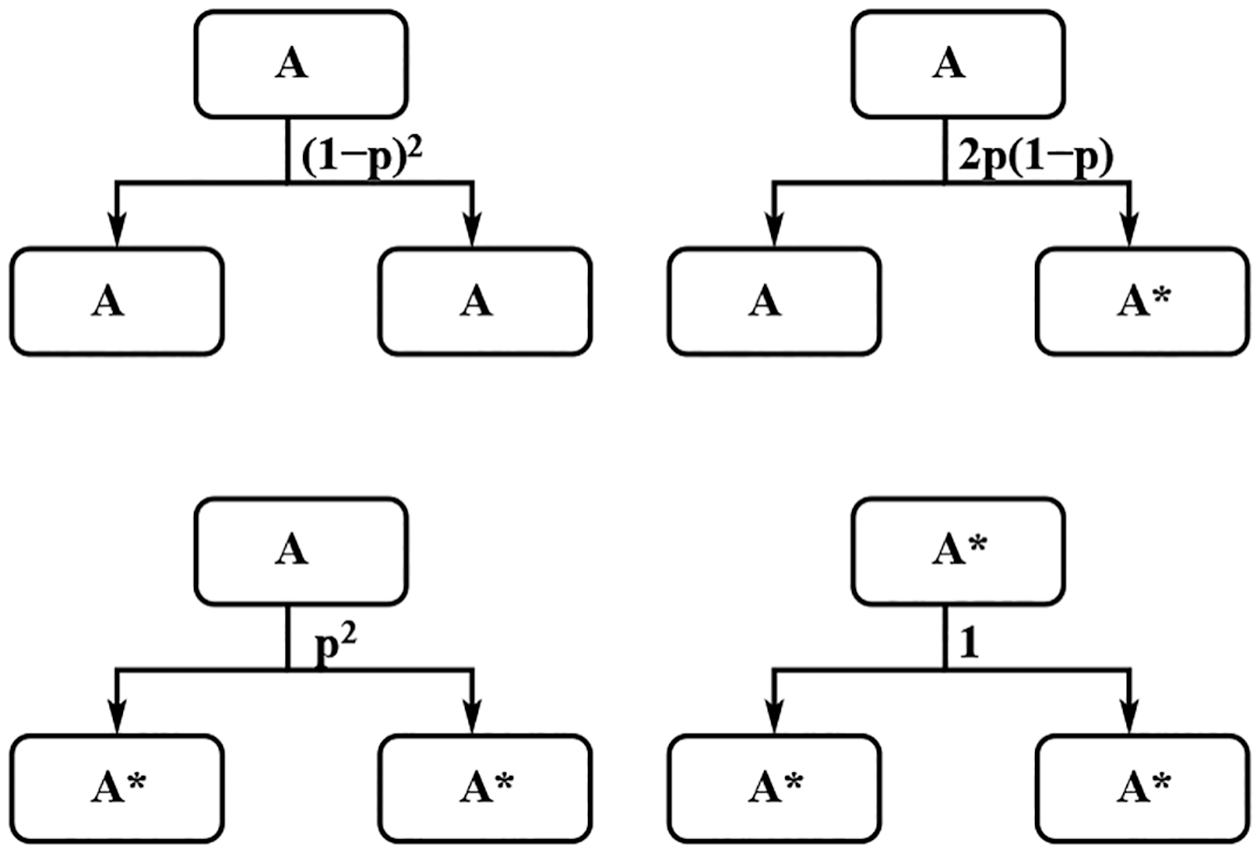
The dynamics of Stem A cells and stem mutant A* cells in the MiST cell division with green light scenario.

**Figure 6.**
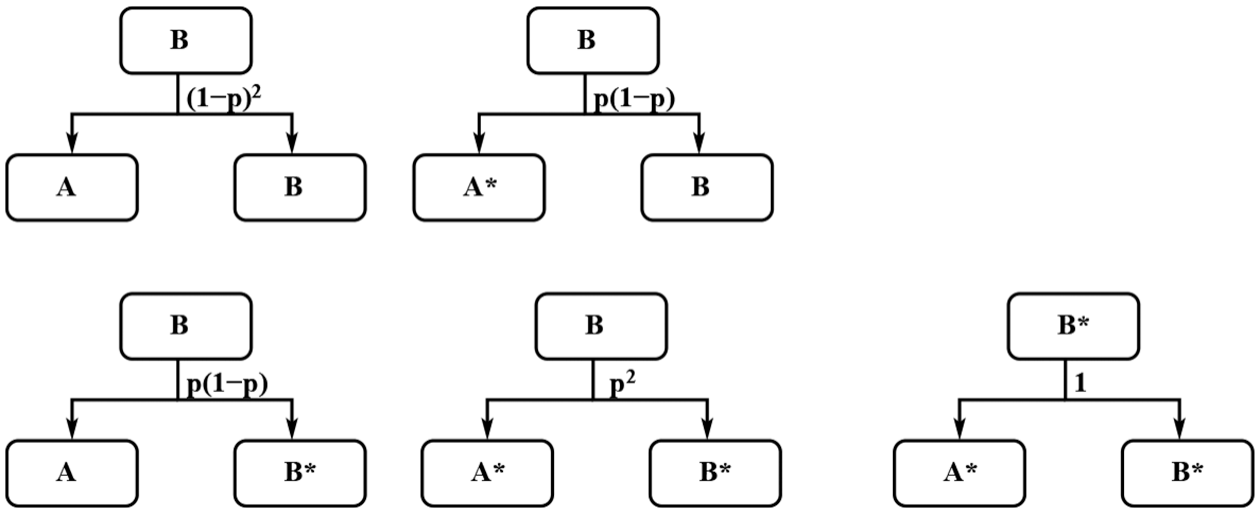
The dynamics of factory cells, and mutant factory cells in the MiST cell division with green light scenario.

For the green light scenario, the dynamics of these cells are described in Figures 5 and 6. The green light scenario can be treated as the inverse between the stem cells and factory cells in the red light case. We use the same notation *p* for the possibility that one of the daughter cells is mutant. One stem cell could produce two stem cells with probability (1 − *p*)^2^, one stem cell and one mutant stem cell with probability 2*p*(1 − *p*), and two mutant stem cells with probability *p*^2^. One factory cell will produce one stem cell and one factory cell with probability (1 − *p*)^2^, one mutant stem cell and one factory cell with probability *p*(1 − *p*), one stem cell and one mutant factory cell with probability *p*(1 − *p*), and one mutant stem cell and one mutant factory cell with probability *p*^2^. One mutant factory cell produces one mutant factory cell and one mutant stem cell with probability 1. One mutant stem cell produce two mutant stem cells.

Let *A*_*g*_(*t*) and $A_{g}^{*}(t)$ represent the numbers of stem cells and mutant stem cells at time t, and *B*_*g*_(*t*) and $B_{g}^{*}(t)$ represent the numbers of factory cells and mutant factory cells at time t. The following ordinary differential equations can be used to model the green light scenario

(2.14) $$\{\begin{array}{l} \frac{dB_{g}(t)}{dt}=r_{bg}B_{g}(t)C_{g}(t)-mB_{g}(t)\\ \frac{dB_{g}^{*}(t)}{dt}=c_{bg}^{*}(t)B_{g}(t)C_{g}(t)-mB_{g}^{*}(t)\\ \frac{dA_{g}(t)}{dt}=\left(r_{ag}A_{g}(t)+c_{bag}B_{g}(t)\right)C_{g}(t)-mA_{g}(t)\\ \frac{dA_{g}^{*}(t)}{dt}=\left(r_{ag}^{*}A_{g}^{*}(t)+c_{ag}A_{g}(t)+c_{bag}^{*}B_{g}(t)+c_{bag}^{**}B_{g}^{*}(t)\right)C_{g}(t)-mA_{g}^{*}(t) \end{array}$$

with $C_{g}(t)=1-\left(B_{g}(t)+B_{g}^{*}(t)+A_{g}(t)+A_{g}^{*}(t)\right)/K$. Similarly, we can calculate the growth rates in the above model which are summarized in the Table 1.

#### 2.2.3. Green light star scenario

**Figure 7.**
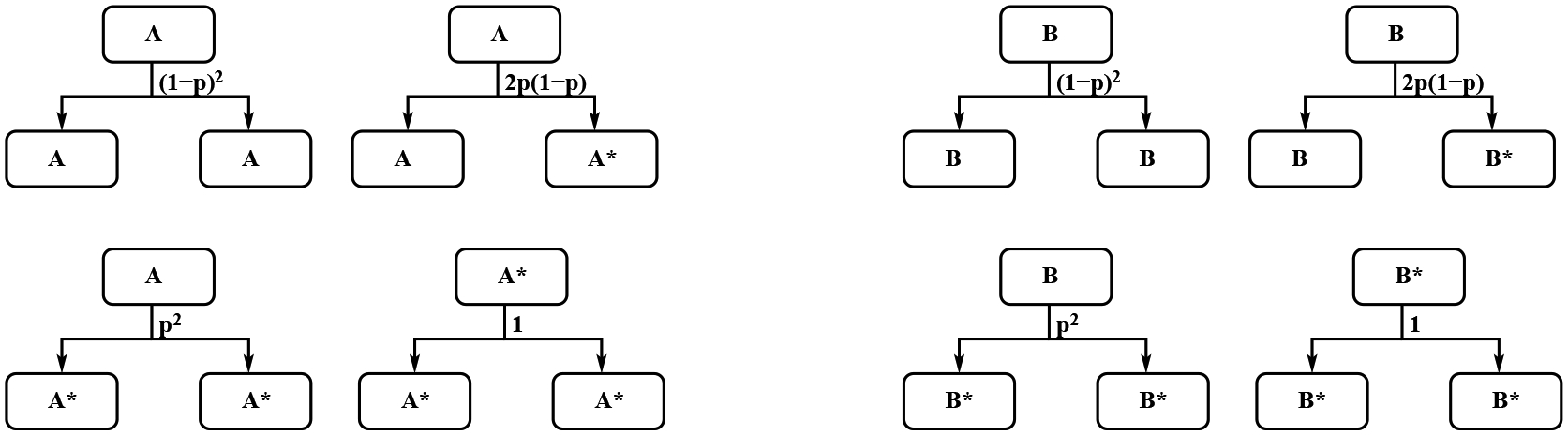
The dynamics of Stem *A* cells, stem mutant A* cells, factory cells, and mutant factory cells in the MiST cell division with green light star scenario.

It is possible that in the green light case, the factory cells and mutant factory cells have the same dynamics as the standard cell division instead of producing stem cells and mutant stem cells. But the stem cells and mutant stem cells will follow the dynamics in the green light case, see Figure 7. The dynamics in this case can be written as

(2.15) $$\{\begin{array}{l} \frac{dB_{gs}(t)}{dt}=r_{b}B_{gs}(t)C_{gs}(t)-mB_{gs}(t)\\ \frac{dB_{gs}^{*}(t)}{dt}=\left(r_{b}^{*}B_{gs}^{*}(t)+c_{b}B_{gs}(t)\right)C_{gs}(t)-mB_{gs}^{*}(t)\\ \frac{dA_{gs}(t)}{dt}=r_{ags}A_{gs}(t)C_{gs}(t)-mA_{gs}(t)\\ \frac{dA_{gs}^{*}(t)}{dt}=\left(r_{ags}^{*}A_{gs}^{*}(t)+c_{ags}A_{gs}(t)\right)C_{gs}(t)-mA_{gs}^{*}(t) \end{array}$$

where $C_{gs}(t)=1-\left(B_{gs}(t)+B_{gs}^{*}(t)+A_{gs}(t)+A_{gs}^{*}(t)\right)/K$. The parameters *r*_*b*_, $r_{b}^{*}$, and *c*_*b*_ are defined in (2.3), (2.6), and (2.5), respectively. We can derive that

$$r_{ags}=\frac{\text{ln}(2(1-p))}{\tau_{b}^{*}},r_{ags}^{*}=\frac{\text{ln }2}{\tau_{b}^{*}},c_{ags}=\frac{2p\left(r_{ags}^{*}-r_{ags}\right)}{e^{r_{ags}^{*}\tau_{b}^{*}}-e^{r_{ags}\tau_{b}^{*}}}.$$

For the basic model, red light model, and green light model, as long as *r*_*b*_ > *m* and $r_{b}^{*}>r_{b}$, the mutant cells, including A* and B*, will dominant and factory cells will die out. But the red light and green light models will slow down the speed that the factory cells become extinct in the vessel.

#### 2.2.4. Red-green light scenario

For the red-green light scenario, the dynamics are the combination of the red light scenario and the green light scenario described above. The switch between these two cycles is decided by a given threshold value *q* ∈ (0, 1). If the percentage of factory cells to the total number of cells in the vessel is above the threshold value, the vessel is kept in the red light cycle, and if the percentage of factory cells drops below the threshold, the vessel is kept in the green light cycle until above the threshold again. This design allows the vessel to regenerate factory cells in the green light cycle, and then increase production of factory cells in the red light cycle. The threshold value is kept relatively high so the constant removal or harvesting of the vessel can be kept as mostly factory cells. Note that switching between light cycles cannot occur continuously as cells need time to adapt to a particular light cycle. To account for this, the vessel is held in a light cycle for a time length of at least one doubling time of factory cells.

#### 2.2.5. Red-green light star scenario

For the red-green light star scenario we use the same dynamics as the red light scenario and green light star scenario described above. Again we change between the two cycles according to a threshold value q* ∈ (0, 1). If the percentage of factory cells to the total number of cells in the vessel is above the threshold value, the vessel is kept in the red light cycle, and if the percentage of factory cells drops below the threshold, the vessel is kept in the green light star cycle until above the threshold again. This switch allows the vessel to regenerate factory cells in the green light star cycle, and then increase production of factory cells in the red light cycle. Once again the threshold value is kept relatively high so the constant removal or harvesting of the vessel can be kept as mostly factory cells, and is kept at *q* = 0.9, or 90% of the vessel is assumed to be factory cells. Similarly, the vessel is held in a light cycle for a time length of at least one doubling time of factory cells.

## 3. Numerical simulations

### 3.1 Numerical simulations for models

**Figure 8.**
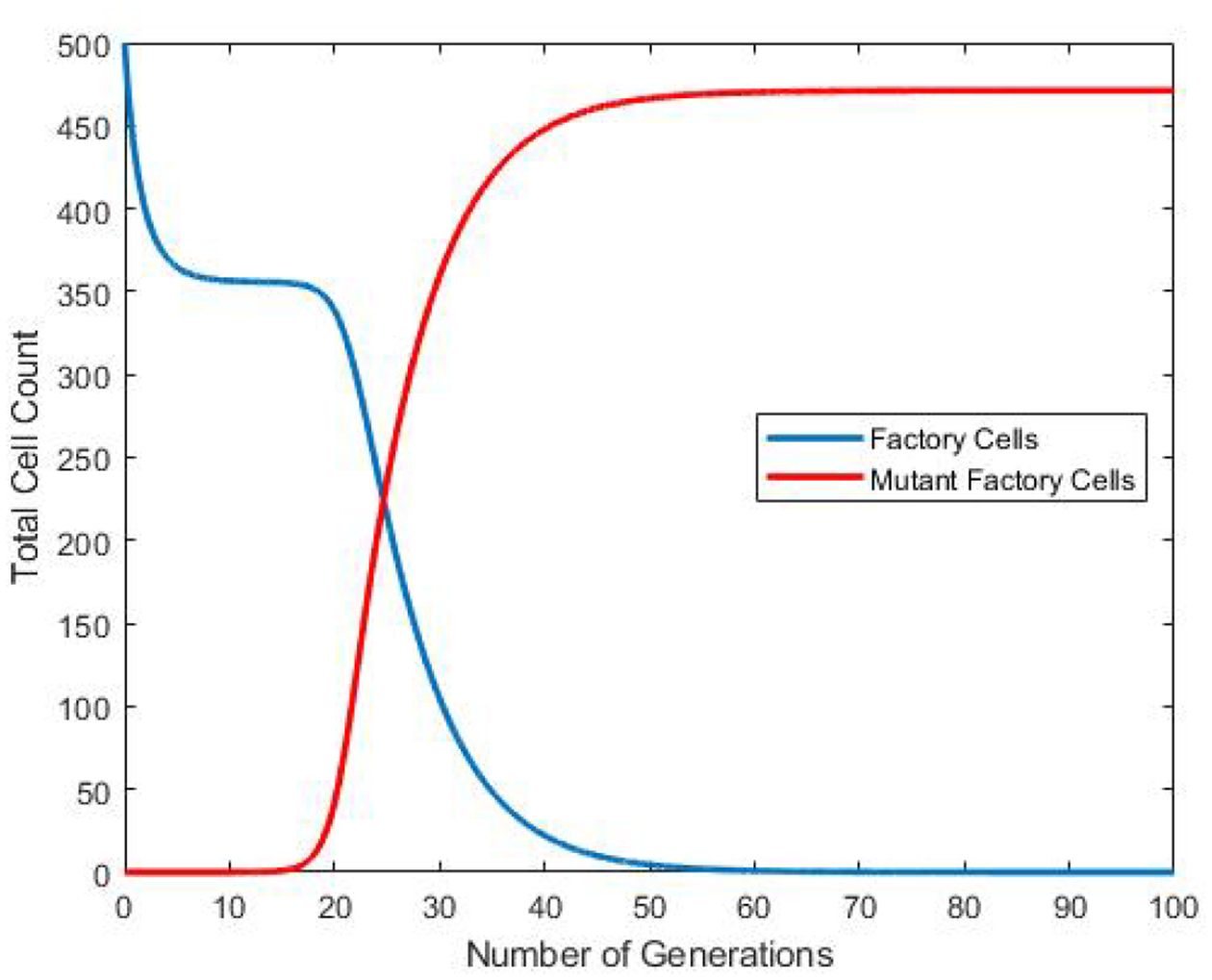
Number of Cell Generations vs. Total Cell count in the vessel for the base model. Shown are the populations of factory cells and mutant factory cells. After several generations pass, the mutant population takes over. A generation time is normalized to be the doubling time of a factory cell.

The goal in creating the base model (2.1) was to provide a means of simulating the population dynamics of conventional cell cultures in continuous flow bioreactor vessels. For all the simulation, the mutant parameter *p* is taken as 10^−6^. Figure 8 shows the predicted outcome given the standard values for the rates of factory cell and escape mutant cell division used throughout this work. As predicted by other mathematical models and confirmed in empirical tests [13], the population of nonproductive escape mutants, whose doubling rate is 5-fold faster than factory cells, eventually overtakes the vessel. The changes in the number of cells in the vessel over time are attributed to changes in the overall growth rate of the culture. Prior to escape mutant take-over, the growth rate of the culture is relatively slow and the constant exchange of volume during continuous flow has a stronger effect on reducing the total number of cells.

**Figure 9.**
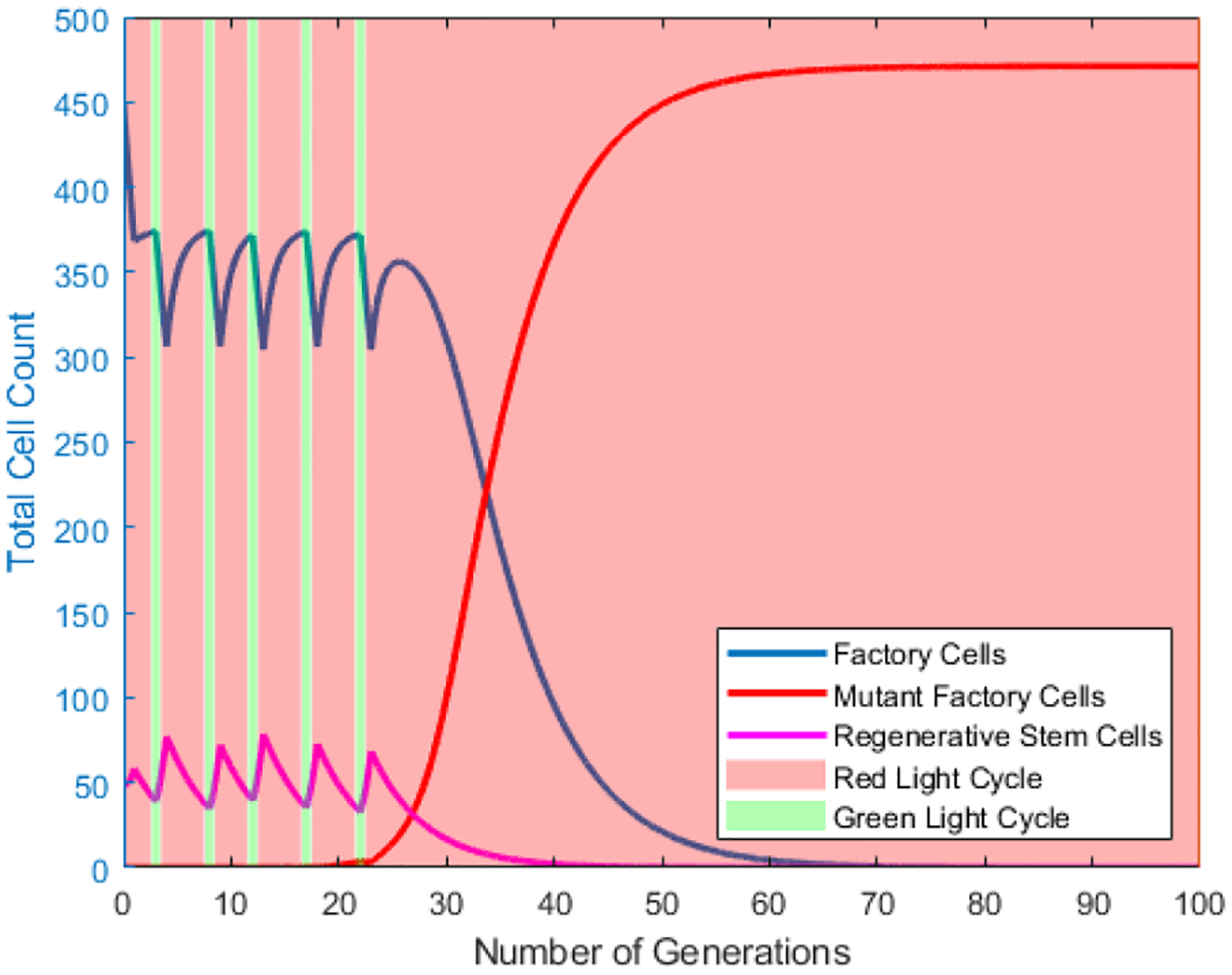
Plot of Number of Generation vs the Total Cell Count in the vessel for various cell populations in **the red-green light** model. Red shaded sections correlate to the red light cycle and green shaded to the green light cycle. Value of *q* = 90%. Value of *m* = 20%. Cell division ratio = 5. Initial Regenerative stem cell population = 10% of the vessel.

**Figure 10.**
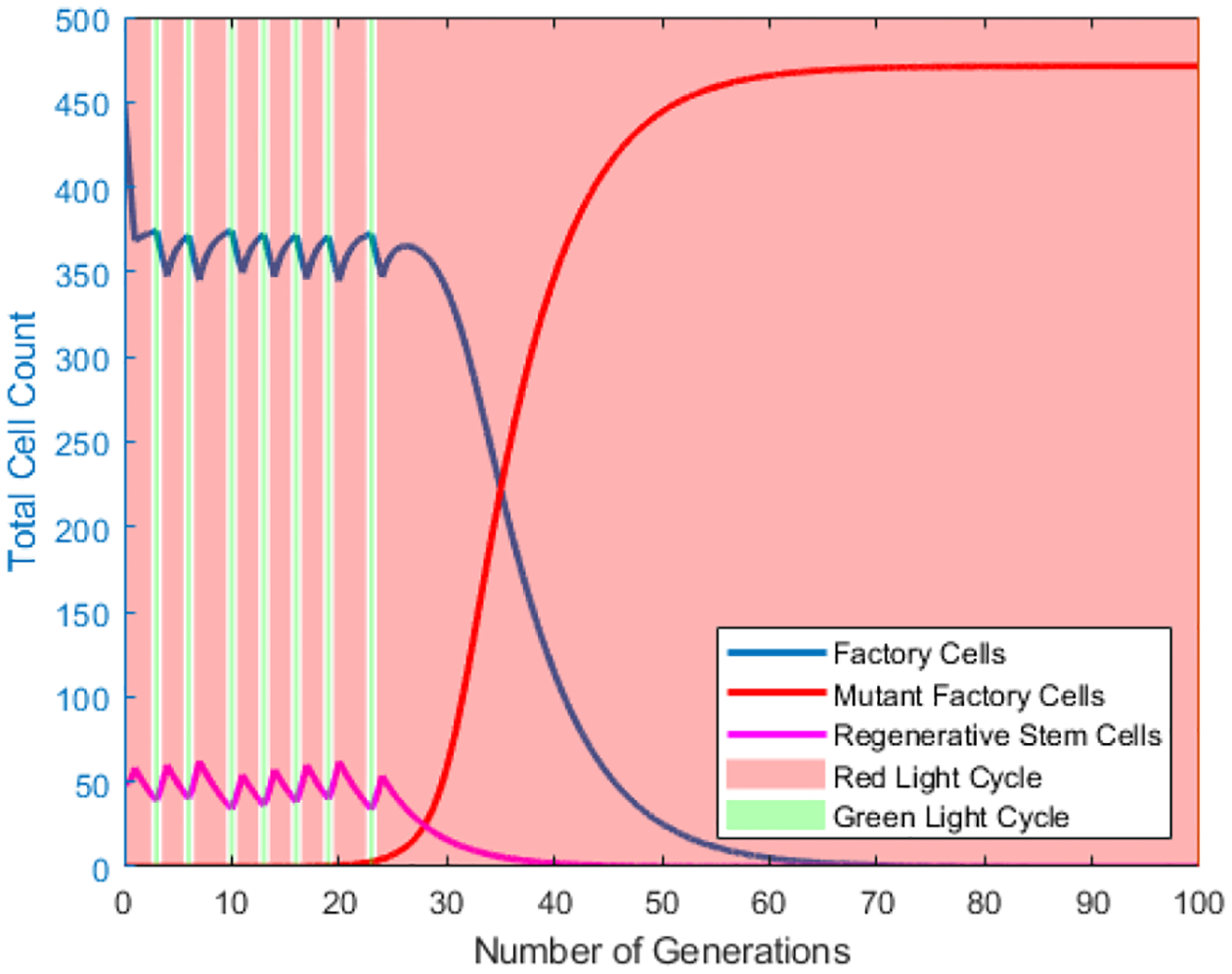
Plot of Number of Generation vs the Total Cell Count in the vessel for various cell populations in the **red-green light star** scenario. Red shaded sections correlate to the red light cycle and green shaded to the green light cycle. Value of *q* = 90%. Value of *m* = 20%. Cell division ratio = 5. Initial Regenerative stem cell population = 10% of the vessel.

Numerical simulations of the base model (2.1) with only factory cells and mutant daughter cells were compared to the MiST red-green light model and the red-green light star model. Simulations were run with several parameters held fixed based on the physical limitations of the proposed vessel. The percentage of factory cells is held throughout all simulations to be as close to 90% when possible for harvesting purposes, as lower values would impose physical difficulties for product harvesting. This corresponds to the parameter *q* for switching between light cycles. Parameters which were varied were the constant rate of removal from the vessel for harvesting *m* with units of volume of the vessel per doubling time of mutant factory cells or, $K/\tau_{b}^{*}$, and an index created as the cell division ratio between factory cells and stem cells and mutant cells $\tau_{b}/\tau_{b}^{*}$. Changes between light cycles happen when the total percentage of factory cells falls below a threshold *q*, which is maintained to be 90%. As the vessel progresses, the threshold value cannot be kept above 90% indefinitely. Once this occurs, the vessel is set to the red light cycle and allowed to finish producing until complete takeover by mutant cells. The vessel is left in the red light cycle to finish, as the red light cycle is the cycle for the highest production of factory cells. The parameters for rate of harvesting and cell division ratio in Figures 9 and 10, are the same as those in Figure 8.

The vessel is given a set of initial conditions. The probability of mutation *p* is fixed, and the carrying capacity of the vessel *K* is the maximum size of a cell population which can be contained within the vessel. A preliminary simulation is run of both the red-green light model and the red-green light star model to showcase the change in the different cell populations over time. As the simulation progresses the population of factory cells oscillates with the current light cycle. The red light cycle are periods of increased factory cell division, where the green light cycle are periods of factory cell regeneration by means of stem cells. In these preliminary simulations, initial vessel conditions are chosen to show how change in light cycles affects the different cell populations. During green light stimulation, when the rate of stem cell doubling is 5-fold faster than the factory cell division rate. The directions of the population shifts are reversed under red light stimulation, though the rate of change is somewhat slower. This is because the number of stem cells remains constant under red light, while the number of factory cells increases at a relatively slow rate due to biological burdens associated with product synthesis [10–12]. Ultimately, the population fraction of escape mutants climbs to 100%, marking the end of production.

**Figure 11.**
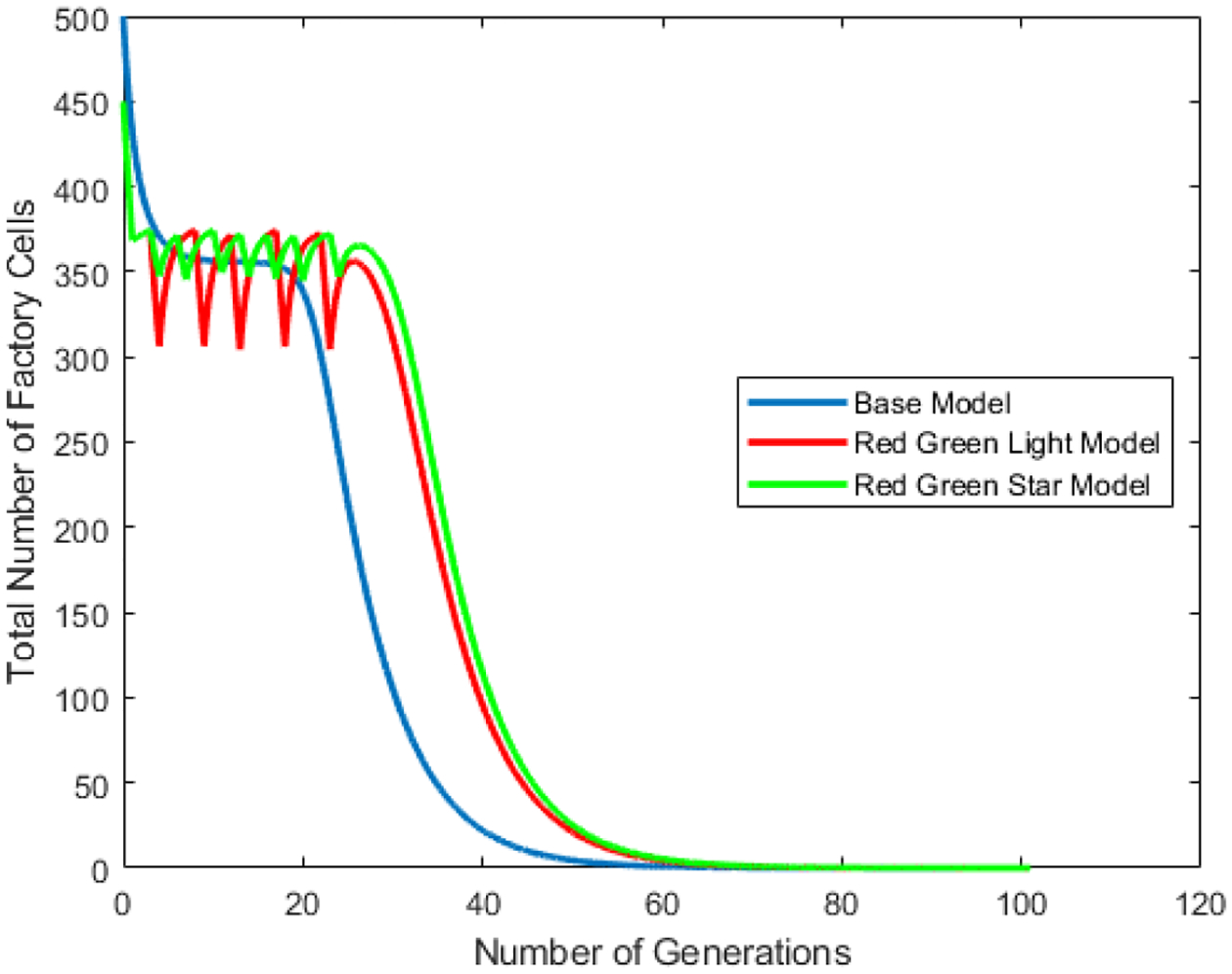
Number of Cell Generations vs. Total Number of Factory Cells in the vessel for the base model. Shown are the populations of factory cells for the base model, red-green light model, and red-green light star model. After several generations pass, the factory cell population approaches to zero, and is completely taken over by mutant factory cells.

**Figure 12.**
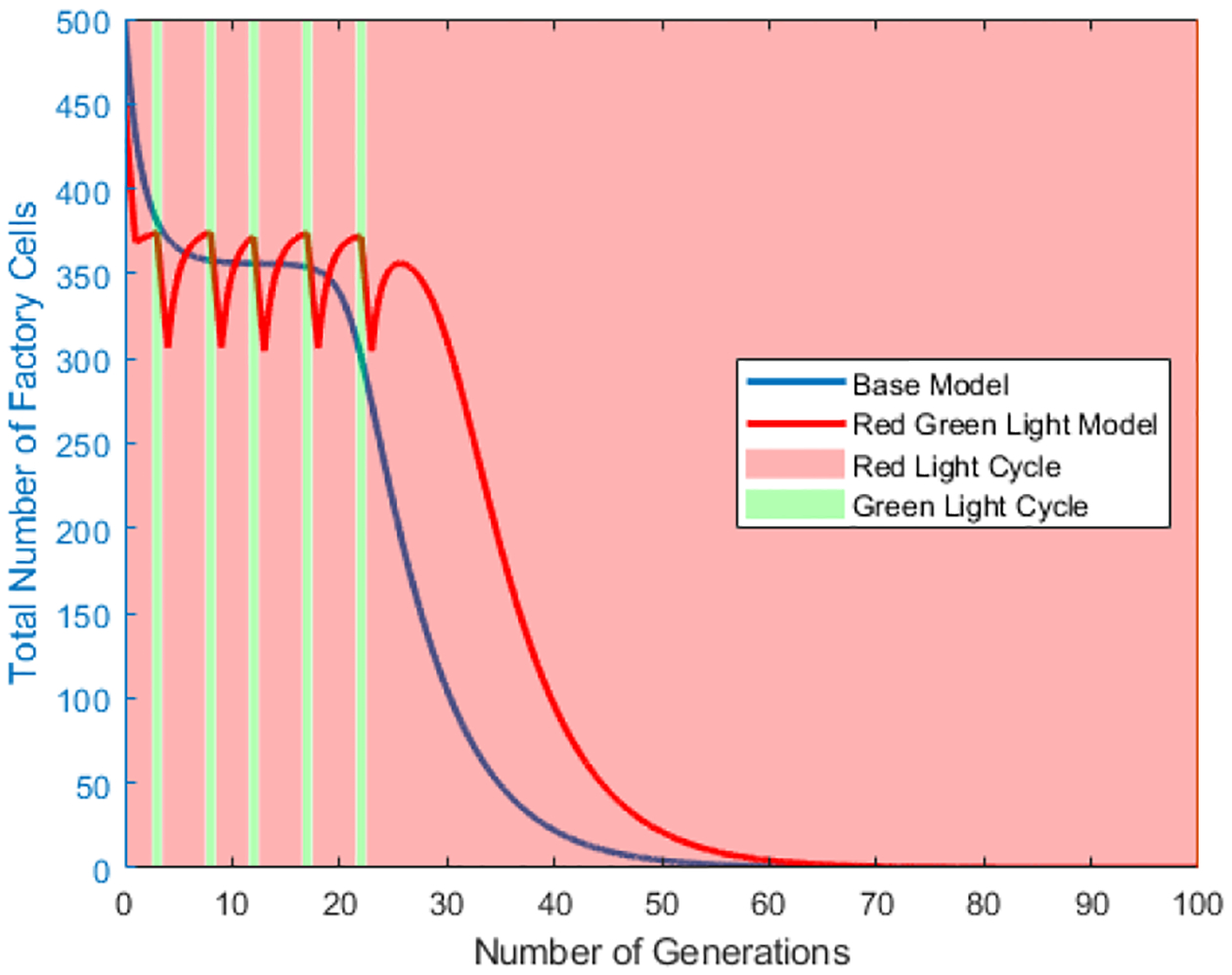
Plot of Number of Generation vs the Total Cell Count in the vessel for factory cell population in **the red-green light** scenario. Red shaded sections correlate to the red light cycle and green shaded to the green light cycle. Value of *q* = 90%. Value of *m* = 20%. Cell division ratio = 5. Initial Regenerative stem cell population = 10% of the vessel.

**Figure 13.**
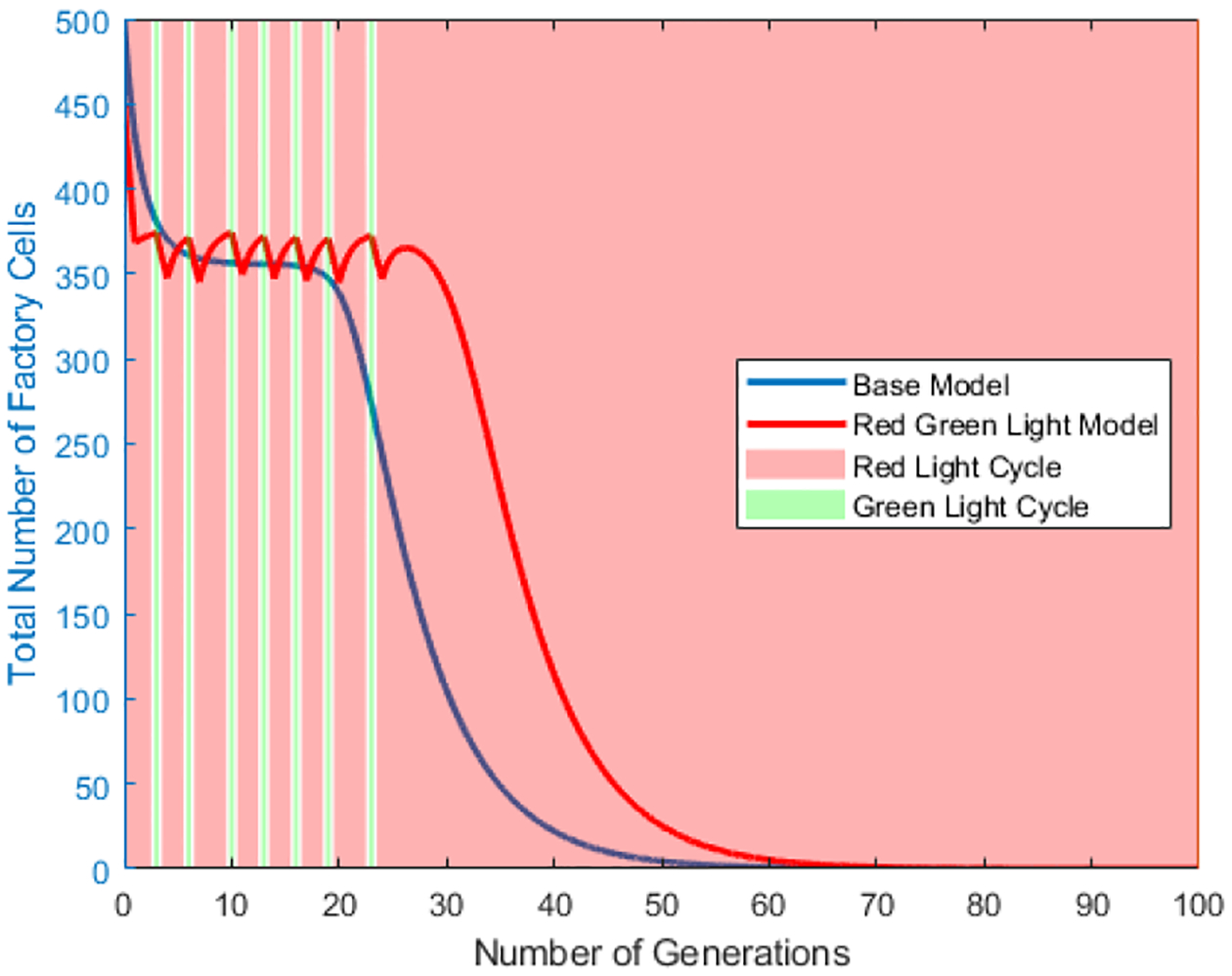
Plot of Number of Generation vs the Total Cell Count in the vessel for factory cell population in **the red-green light star** Scenario. Red shaded sections correlate to the red light cycle and green shaded to the green light cycle. Value of *q* = 90%. Value of *m* = 20%. Cell division ratio = 5. Initial Regenerative stem cell population = 10% of the vessel.

The red-green light and red-green light star scenarios are then compared to the base model using the same set of initial parameters to determine if there is a set of parameters under which either the red-green light and red-green light star scenarios can increase the number of factory cells. Figure 11 shows the population of factory cells for the base model, the red green light model and the red green light star model over time. Figures 12 and 13 break Figure 11 into the red green light and red green light star models respectively, and compare them to the base model while showing the different light cycling.

We use the following index

$$B_{Har}=\int_{0}^{n\tau_{b}}m\frac{B(t)}{N(t)}dt$$

to measure the difference among the red-green light model, red-green light star model, and the base model. Where *B*_*Har*_ is the total percentage of factory cells which were harvested through the duration of the simulation, *n* is the number of generations of cell divisions in the simulation, *m* is the constant flow out of the vessel, and *B*(*t*)/*N*(*t*) is the fraction of the vessel currently occupied by factory cells. We then use this index to plot several surfaces which show how ranging the ratio between cell division times and the rate of constant harvesting from the vessel influences the the total number of factory cells which are harvested from the simulation.

### 3.2. Sensitivity analysis

A sensitivity analysis was conducted to better understand each parameters impact on the outcome of the numerical simulation for different models. Parameters which were held fixed were, *q*, the threshold value for switching light cycles, because of physical limitations on harvesting, and the initial cell populations in the vessel when the simulation began, as this parameter shows little change in the results of the simulation. Then the red green light and red green light star model were plotted as a surface with varying ratio of factory cell division, $\tau_{b}/\tau_{b}^{*}$, and the rate of flow out of the vessel or cell harvesting, *m*. The index of *B*_*Har*_ was then used to compare each of the red-green light and red-green light star models to the base model. These plots are shown in Figures 15 and 16, respectively.

**Figure 14.**
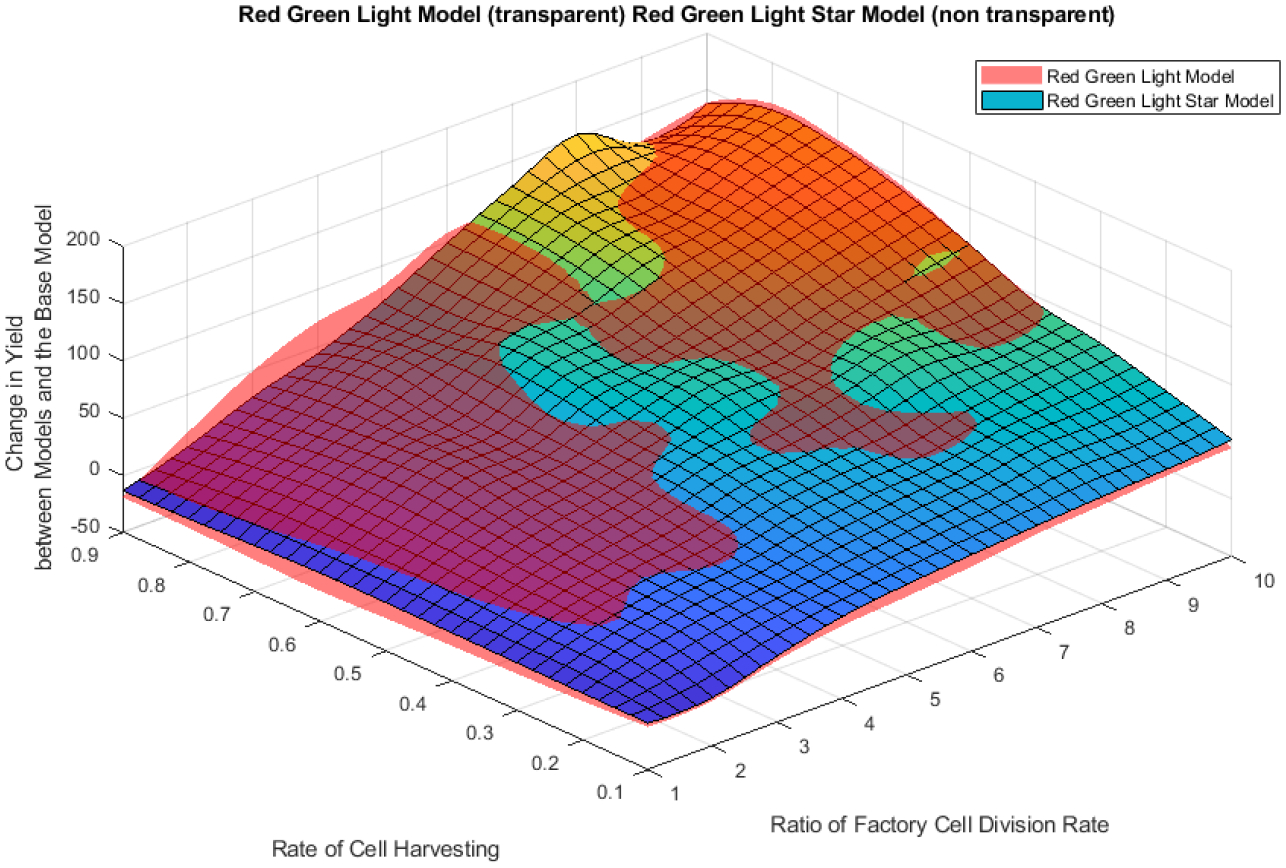
Plot of Rate of Factory Cell Division Rate and Cell Harvesting Rate to the Change in Yield of Model compared to the Base Model. Value of *q* = 90%. Value of m ranges from 10% to 90%. Cell division ratio ranges from 1 to 10. Initial Regenerative stem cell population = 10% of the vessel.

**Figure 15.**
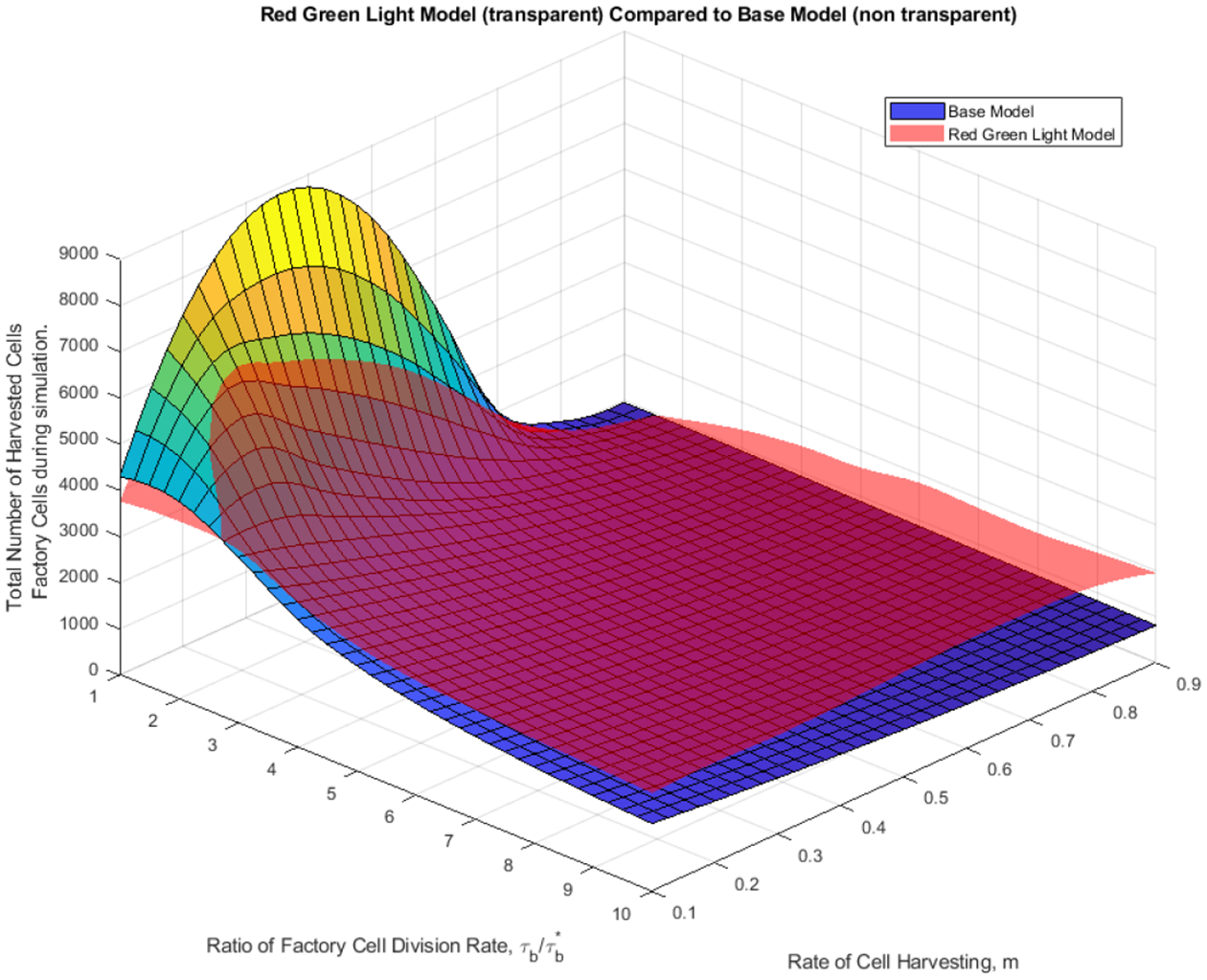
Plot of Rate of Factory Cell Division Rate and Cell Harvesting Rate to the Total Number of Harvested Factory Cells during the red-green light model. Value of *q* = 90%. Value of *m* ranges from 10% to 90%. Cell division ratio ranges from 1 to 10. Initial Regenerative stem cell population = 10% of the vessel.

**Figure 16.**
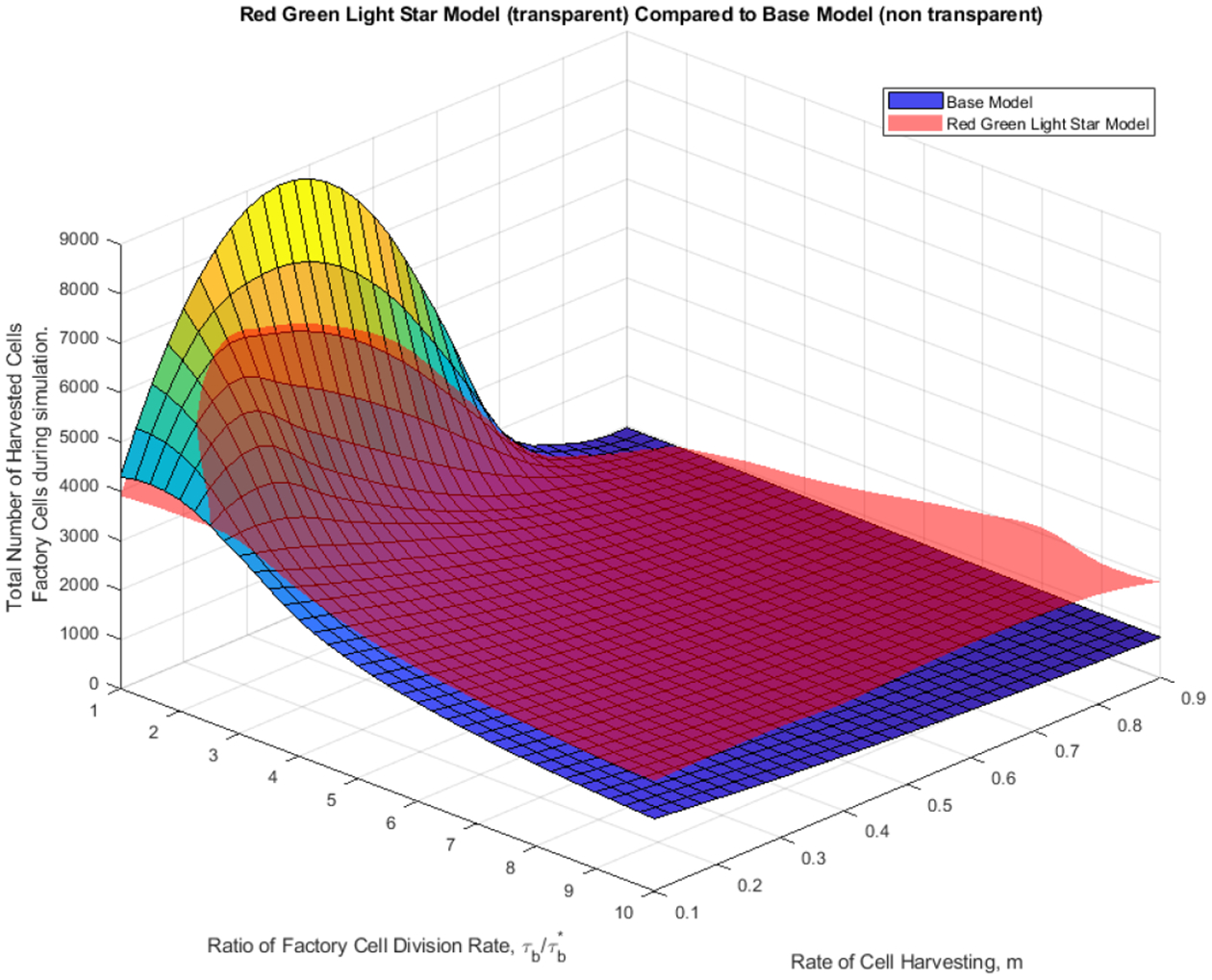
Plot of Rate of Factory Cell Division Rate and Cell Harvesting Rate to the Total Number of Harvested Factory Cells during the red-green light star model. Value of *q* = 90%. Value of *m* ranges from 10% to 90%. Cell division ratio ranges from 1 to 10. Initial Regenerative stem cell population = 10% of the vessel.

From these plots we can immediately infer the base model only out produces the red-green light model and red-green light star model when the cell division ratio between the factory cells and mutants cells, $\tau_{b}/\tau_{b}^{*}$, is larger than one. Intuitively this makes sense, as there is no biological burden placed on the factory cells, and they can divide as fast as the mutant cells. At values of cell division ratios which are greater than one, we see both the red-green light model and red-green light star model out produce the base model for all values of cell harvesting rates. To investigate how the red-green light model and red-green light star model compare to each other, we create an index of change in yield comparing each model to the base model. From Figure 14, we can see neither the red-green light model or red-green light star model is best over all sets of cell division ratios and harvesting rates. Which model produced a better yield is determined based on these initial parameters. Again, yield is showing as negative at a division ratio of 1, but as the division ratio increases, the yield from the red-green light model and red-green light star model always increases over the base model.

### 3.3. Discussion

From these simulations we are able to infer when the ratio of factory cell to mutant cell division is near 1, the base model out performs both the red-green light model and red-green light star model. Intuitively this makes sense, as the lower the ratio of division between the cell types, the less biosynthetic burden is placed on the factory cells compared to the mutant cells. When this ratio is increased, this shows the effect of the biosynthetic burden placed on the factory cells to create a product. We see that both the red green light and red green light star models show improvement over the base model for all values of cell division ratios greater than 2. We conclude for every set of parameters where the biosynthetic burden placed on the factory cells causes them to divide at least half as slowly as the mutant cells, the red green light and red green light star models will produce a high yield of cell product. The flow into and out of the vessel, denoted as *m*, shows little fluctuation in the number of total harvested cells across the ranges of 10 to 90%. Varying initial population in the vessel between factory cells and regenerative stem cells shows changes in the total harvested cells of 1–2%, indicating how the vessel is initialized has little effect on the outcome of product. This is explained by the vessel having an initial period of red light time where the cell populations always approach some initial carrying capacity for each particular cell population based on the parameter *q*, which remains fixed due to physical constraints.

Whether the red green light or red green light star model produces a higher yield of harvested cells cannot be determined universally across all parameters sets. Both the red green light and red green light star model improve the amount of harvested cells when compared to the base model at cell division ratios greater than 2, however neither the red-green light nor red-green light star model outperforms the other globally over the parameter space.

Based on the outcomes of these models, we predict that MiST could be used to achieve a substantial increase in product yields from continuous flow bioreactors. Comparing standard microbial cell culture to MiST cell cultures under a standard set of conditions predicted an increase of at least 29.7%. Notably, our models also predict that the relative benefit of MiST is greatest when product producing “factory” cells suffer reduced division rate. Thus, MiST promises the most benefit for difficult biosynthetic processes and toxic products. As bioengineering becomes more complex, we can expect an increasing number of difficult syntheses. Many toxic products are already known. Some of these are cell permeable, so the benefit to stem cells would be limited by membrane diffusion rate. Many products are not cell permeable, such as proteins and peptides or hydrophobic molecules such as those used in biodiesel. In our models, committing factory cells to production was not much better than allowing them to change back into stem cells. But these models did not take into account the possibility that stem cells derived from factory cells mar retain some biosynthetic burden. If this case, it is logical to predict that the committed model will fare better, depending on the carry-over of that burden. A remaining question is whether MiST could benefit fed-batch bioreactors, which are more common. Given that fed-batch bio-processes suffer from the same problems related to genetic stability, it is reasonable to conclude that MiST would also substantially benefit this type of bioprocess. Here, production was predicted to increase by 1 to 2-fold.

## 4. Conclusion

The goal of this study was to model the behavior of cell cultures in continuous-flow bioreactors that are controlled using MiST culturing methods. A central concept of MiST is that a sub-population of “stem cells” can be established and maintained within a culture, and the relatively rapid rate of cell division of this cell type can be useful for generating large numbers of new, product-producing “factory cells”. Rapid growth of the factory cell population is hypothesized to provide effective competition against overpopulation by “escape mutants” that have lost the ability to make product. A question is whether the rapid generation rate of new factory cells could be sufficient to delay overpopulation by escape mutants and thereby prolong the production phase.

Our models assume a number of significant elements related to bioreactor design. First, they relate specifically to continuous flow bioreactors, which are amenable to the continuous type of mathematical modeling we have used, wherein the outcome is a steady-state solution. We chose to model continuous flow bioreactors because this biofementation strategy offers great potential for low-cost, broadly scalable production [22], yet widespread implementation is hindered by the risk of culture overpropulation by rapidly dividing escape mutants [13]. Other bioreactor designs, including single-run and fed-batch biofermentation, are not addressed in this study, although these bioprocesses face similar problems with escape mutant overpopulation, particularly at large scales.

Another element of bioreactor design assumed in our models is the ability to use light illumination to control the cells’ genetic circuitry. The walls of the biofermentation vessel must be transparent to light, as is the case for glass-walled bioreactors and disposable bioreactors with thin plastic walls, or a light source must be inserted directly into the vessel if its walls are opaque. A related assumption of our models is that light exposure can be modified in accordance with the changing percentage of stem cells in the vessel. Thus, there must be a method for measuring the percentage stem cells in real-time. Since stem cells can be programmed to express a red fluorescent marker, such measurements are achievable with flow cytometry or fluorescence microscopy [21], but the required instrumentation would have to be added at additional cost.

Our models predict that MiST culturing methods can produce higher yields than conventional cell culturing methods in light-controllable continuous flow bioreactors. Sensitivity analyses indicate that this can be achieved over a broad range of parameter space including, harvesting flow from the vessel *m*, initial vessel populations, and conditions for changing between light cycles *q*, and that MiST generates higher relative increases in yield as the growth rate of factory cells slows compared to stem cells and escape mutants. Thus, our models support the hypothesis that MiST can be useful when product production has significant inhibitory effects on factory cell growth. Notably, our models show that the presence of stem cells delays, but does not prevent overpopulation by escape mutants. This is because escape mutants produce two rapidly dividing escape mutant progeny cells with every cell division, whereas stem cells produce one rapidly dividing stem cell and one slowly dividing factory cell during periods of red light stimulation.

As strain bioengineering becomes more sophisticated, synthetic biologists are gaining the ability to synthesize a wider number of chemical products, including many whose synthesis will have negative effects on factory cell growth. Some products readily diffuse across the cell membrane and would affect the growth of factory cells and stem cells nearly equally, thereby canceling the advantages of MiST. Other products are retained within factory cells due to hydrophobicity, insolubility, or other chemical properties, and these are more promising candidates for MiST bioprocessing. Further, growth inhibition is often a consequence of metabolic burdens placed upon the cell during the synthesis of enzymes in the biosynthetic pathway, not the toxicity of the end product itself. In these cases, growth inhibition is specific to the factory cells, and we predict that yields will be increased by using MiST culturing techniques.

Our sensitivity analyses also predicted that MiST culturing would generate higher yields relative to traditional cell culturing when the flow rate of the bioreactor is increased. This is sensible because higher flow rates mean faster influx of fresh nutrients and therefore more space within the vessel for cells to grow, leading to more cell divisions. Rapid cell division amplifies the rate of change in population frequencies when there are different growth rates for factory cells and escape mutants. Again, the problem of rapidly dividing escape mutants appears to be mitigated by using MiST to maintain a population of rapidly dividing, factory cell producing stem cells.

With regard to flow rates, an advantage of continuous flow bioreactors is that product can be harvested from the outflow as soon as it is removed from the bioreactor. By contrast, product must remain in the system for a longer period of time when culture media is harvested from singe-run or batch-fed systems. Given that MiST is more advantageous at higher flow rates, the results suggest that this technology could be particularly useful for boosting the yields of unstable chemical products.

## Figures and Tables

**Table 1.** The definition of parameters in the green light model (2.14).

Parameter	Definition	Value
*r*_*bg*_	The intrinsic growth rate of factory cells	$\frac{\text{ln}(1-p)}{\tau_{b}}<0$
$c_{bg}^{*}$	The growth rate of mutant factory cells due to factory cells	$-\frac{\text{ln}(1-p)}{\tau_{b}}$
*r*_*ag*_	The intrinsic growth rate of stem cells	$\frac{\text{ln }2(1-p)}{\tau_{b}^{*}}$
*c*_*bag*_	The growth rate of stem cells due to factory cell	$(1-p)\frac{r_{bg}-r_{ag}}{e^{r_{bg}\tau_{b}}-e^{r_{ag}\tau_{b}}}$
$r_{ag}^{*}$	The intrinsic growth rate of mutant stem cells	$\frac{\text{ln }2}{\tau_{b}^{*}}$
*c*_*ag*_	The growth rate of mutant stem cells due to stem cells	$\frac{2p\left(r_{ag}^{*}-r_{ag}\right)}{e^{r_{ag}^{*}\tau_{b}^{*}}-e^{r_{ag}\tau_{b}^{*}}}$
$c_{bag}^{*}$	The growth rate of mutant stem cells due to factory cells	$-\frac{\text{ln}(1-p)}{\tau_{b}}$
$c_{bag}^{**}$	The growth rate of mutant stem cells due to mutant factory cells	$\frac{\text{ln }2}{\tau_{b}^{*}}$
